# Recommendations from the ASCO Academic Global Oncology Task Force

**DOI:** 10.1200/GO.20.00497

**Published:** 2020-11-05

**Authors:** Julie R. Gralow, Fredrick Chite Asirwa, Ami Siddharth Bhatt, Maria T. Bourlon, Quyen Chu, Alexandru E. Eniu, Patrick J. Loehrer, Gilberto Lopes, Lawrence N. Shulman, Julia Close, Jamie Von Roenn, Michal Tibbits, Doug Pyle

**Affiliations:** ^1^ University of Washington/Fred Hutchinson Cancer Research Center, Seattle, WA; ^2^ International Cancer Institute, Eldoret, Kenya; ^3^ Stanford University, Stanford, CA; ^4^ Instituto Nacional de Ciencias Médicas y Nutrición Salvador Zubirán, Mexico City, Mexico; ^5^ Louisiana State University Health Sciences Center, Shreveport, LA; ^6^ Oncology Institute Prof Dr I. Chiricuta, Cluj-Napoca, Romania; ^7^ Indiana University Simon Cancer Center, Indianapolis, IN; ^8^ University of Miami, Miami, FL; ^9^ University of Pennsylvania, Philadelphia, PA; ^10^ University of Florida, Gainesville, FL; ^11^ American Society of Clinical Oncology, Alexandria, VA

## Abstract

In recognition of the rising incidence and mortality of cancer in low- and middle-resource settings, as well as the increasingly international profile of its membership, ASCO has prioritized efforts to enhance its engagement at a global level. Among the recommendations included in the 2016 Global Oncology Leadership Task Force report to the ASCO Board of Directors was that ASCO should promote the recognition of global oncology as an academic field. The report suggested that ASCO could serve a role in transitioning global oncology from an informal field of largely voluntary activities to a more formal discipline with strong research and well-defined training components. As a result of this recommendation, in 2017, ASCO formed the Academic Global Oncology Task Force (AGOTF) to guide ASCO’s contributions toward formalizing the field of global oncology. The AGOTF was asked to collect and analyze key issues and barriers toward the recognition of global oncology as an academic discipline, with an emphasis on training, research, and career pathways, and produce a set of recommendations for ASCO action. The outcome of the AGOTF was the development of recommendations designed to advance the status of global oncology as an academic discipline.

## INTRODUCTION

Cancer incidence and mortality are rapidly increasing worldwide. In 2018, the International Agency for Research on Cancer estimated > 18 million new cancer diagnoses and 9.6 million cancer deaths worldwide.^1^ Cancer is an important cause of morbidity and mortality throughout the world, irrespective of a country’s level of economic development. Predictions suggest that by 2030, 13 million people will die as a result of cancer each year, and three quarters of these deaths will be in low- and middle-income countries (LMICs).^2^ Globally, it is estimated that between 2.6 and 4.3 million cancer deaths annually could be avoided with effective prevention and treatment and that LMICs account for the majority of avoidable cancer mortality.^3^ Cancer has become recognized as a global health priority, as evidenced by a series of United Nations high-level meetings on noncommunicable diseases initiated in 2011^4^ and the unanimously adopted WHO 2017 Cancer Resolution.^5^

**CONTEXT**

**Key Objective**
To establish global oncology as a formal discipline, with strong research and well-defined training components.
**Knowledge Generated**
The ASCO Academic Global Oncology Task Force was established to analyze key issues and barriers toward the recognition of global oncology as an academic discipline, with an emphasis on training, research, and career pathways. This publication summarizes a set of 13 recommendations designed to advance the status of global oncology as an academic discipline.
**Relevance**
Global oncology is a necessary academic field to train experts who can think critically about innovative solutions to global cancer care and control, with the tools and support to conduct research and implement change. Collaboration across all sectors of the global health and oncology communities will be required to realize the vision of equity and improved cancer outcomes worldwide.

As science and innovation lead to improvements in cancer prevention, detection, treatment, and palliation, suffering and death as a result of cancer are becoming increasingly concentrated among the poor.^3^ To address growing inequities in cancer care, there is a need to frame cancer control actions and priorities to conform with localized patterns of risk factors and cancer types, available health care infrastructure and resources, and societal and cultural norms. Well-trained scientists equipped with advanced global oncology skills and competencies are needed to solve complex global cancer challenges. This requires effective training programs that include hands-on field experience and high-quality mentoring. For those beyond training, opportunities for a defined career pathway in global oncology, including research funding, institutional support, and metrics for promotion and career advancement, are necessary.^6^

Global oncology is a relatively new field that to date relies heavily on volunteer time, with a paucity of dedicated resources, support, and funding for those looking to pursue global oncology as a career focus. Increasingly, early-career oncology professionals are seeking experiences and training in cancer in a global context. Trainees across oncology subspecialties have shown interest in pursuing training and careers in global health.^7-9^ Despite demonstrated interest in global oncology opportunities, many trainees believe that this opportunity is not offered or available to them. In an ASCO Medical Oncology In-Training Exam survey of medical oncology trainees, 57.1% of respondents would choose to have an in-country global health experience during their fellowship if such an opportunity were offered (ASCO Education, Science & Professional Development Department, personal communication, April 2018). In the same survey, 82.3% of respondents were unaware of in-country global health experiences in their fellowship program. Surveys among trainees in other oncology subspecialties show equally strong interest in global health opportunities and experiences.^10-13^

ASCO has undertaken initiatives designed to enhance its impact globally and expand its international efforts. In a set of ASCO Global Oncology Leadership Task Force recommendations to the ASCO Board of Directors in 2016, the task force outlined a global strategy for ASCO built upon three pillars: innovative global oncology research, quality improvement, and professional development.^14^ The report led to initiatives intended to stimulate and support global oncology, including the launch of *Journal of Clinical Oncology Global Oncology* (JCO GO) in 2015, inclusion of a global health track in 2017 in the education program at the ASCO annual meeting, acceleration since 2016 of the Quality Oncology Practice Initiative (QOPI) and QOPI Certification Program internationally, and the launch of the Global Oncology Young Investigator Award (YIA) in 2018 by ASCO’s Conquer Cancer Foundation (CCF).

The Global Oncology Leadership Task Force also recommended that ASCO promote the recognition of global oncology as an academic field, suggesting that ASCO could serve a role in “the transition of global oncology from an informal field of largely voluntary activity to a formal field with a strong research component and recognized value to oncology training and the practice of oncology.”^14^ The report stated that such an initiative could build on ASCO’s expertise in supporting the professional development of its members domestically and internationally and contribute substantially to addressing the global cancer burden.

As a result, the ASCO Academic Global Oncology Task Force (AGOTF) was formed in 2017 to implement this recommendation and guide ASCO’s contributions toward formalizing the field of global oncology. The AGOTF was tasked with collecting and analyzing key issues and barriers toward the recognition of global oncology as an academic discipline and convening key stakeholders within and outside of the oncology community. The AGOTF was asked to develop a set of written recommendations for ASCO to advance the status of global oncology as an academic discipline, with an emphasis on global oncology training, research, and career pathways. The AGOTF was tasked with identifying issues and obstacles related to the integration of global oncology concepts and skills into the subspecialty training of oncologists, including barriers related to Accreditation Council for Graduate Medical Education (ACGME) requirements for trainees in the United States; identifying skills and competencies required to perform oncology care in underserved and resource-poor settings; outlining a curriculum for global oncology training that could be integrated into a fellowship training program; identifying opportunities to enhance the formal practice of global oncology and expand a well-trained workforce, including support of a rigorous and respected research component; identifying opportunities to support the ongoing professional development of those engaged in global oncology, including mentorship and funding opportunities; and identifying potential career pathways in global oncology and a role for ASCO in supporting the development of these careers and professional opportunities.

## METHODS

The AGOTF was composed of nine members and a liaison from the ASCO Professional Development Committee. It was supported by staff from the ASCO International Affairs and Professional Development Committees. The AGOTF was chaired by J.R.G. and staffed by D.P., ASCO Vice President for International Affairs. To accomplish its work, the AGOTF performed the following activities between 2017 and 2018.

### Conference Calls

The AGOTF conducted conference calls approximately every other month that focused on topics related to its assigned responsibilities.

### Stakeholders Summit

The AGOTF convened a 1-day Global Oncology Stakeholders Summit in April 2018. The summit included leaders in global oncology, with representatives from medical oncology, radiation oncology, surgical oncology, pediatric oncology, and pathology. Attendees included nononcology medical specialties that have successfully incorporated global health, including infectious diseases, and representatives from the National Cancer Institute (NCI) Center for Global Health (CGH), the Centers for Disease Control and Prevention, and the WHO. The summit included current oncology fellows, early-career global oncology faculty, cancer center directors, and training program directors as well as representatives from both high- and low/middle-resource settings. Participants discussed key factors that facilitated other medical disciplines’ incorporation of global health into the training, research, and careers of their specialists; analyzed how these success factors may apply to oncology trainees and professionals, and discussed how ASCO and other oncology specialty organizations can catalyze the application of these success factors to oncology.

### NCI/ASCO Survey on Global Oncology Research and Training Programs in NCI-Designated Cancer Centers

To better understand global oncology activities led by NCI-Designated Cancer Centers (NDCCs), the NCI CGH collaborated with ASCO to conduct the 2018/2019 NCI/ASCO Global Oncology Survey of NDCCs. While this survey was not conducted by the AGOTF, members of the AGOTF participated in the design of the questionnaire. Survey results were reviewed in discussions, which contributed to the AGOTF recommendations. The 70 NDCCs received a two-part survey that focused on global oncology programs and non–National Institutes of Health (NIH)–funded global oncology projects at the NDCCs.

## RESULTS

### Definition of Global Oncology

The AGOTF started by creating a working definition of global oncology for reference throughout its activities and discussions. As defined by the AGOTF, global oncology collaboratively addresses disparities and differences in cancer prevention, care, research, education, and the disease’s social and human impact around the world. It includes a full spectrum of activities ranging from epidemiology to implementation science to public health policy.

### Review of ACGME Requirements and Barriers to Global Oncology

The AGOTF engaged with the ACGME to determine how ACGME rules and regulations could be interpreted to support oncology fellows with an interest in a global oncology experience and career pathway. Opportunities for incorporating international experiences into formal oncology training programs were explored, including fulfilling continuity clinic obligations and onsite mentorship by board-certified oncologists at an international location.

The ACGME Next Generation Accreditation system represents a move toward core competencies rather than the amount of time in training to allow innovation and improvement.^15^ The ACGME’s Program Requirements for Graduate Medical Education in Hematology and Medical Oncology categorizes requirements as being either core or detail.^16^ All training programs must comply with core requirements, but high-performing training programs in good standing are encouraged to innovate with detail requirements. Specifically, this flexibility with the detail requirements for continuity clinics, including the potential for remote instead of onsite mentoring from a board-certified oncologist, represents an opportunity for global oncology rotations and experiences in training programs in good standing.

### Curriculum and Competencies in Global Oncology

The European Society for Medical Oncology and ASCO have published recommendations that outline a standardized medical oncology training curriculum with global competencies required to qualify as a medical oncologist.^17^ The curriculum includes a chapter on cancer care delivery in low-resource environments, with the objective that all medical oncology trainees should be able to understand the challenges of treating cancer with limited resources and in settings with a weaker health care infrastructure. The chapter’s basic outline of awareness, knowledge, and skills is intended for inclusion within the standard curriculum and competencies for all medical oncology training programs.

For a trainee intending to specialize in global oncology, the AGOTF developed a list of knowledge and skills that a trained and certified oncology professional would need to understand and practice as a specialist in global oncology. These competencies were divided into two categories: core global health competencies that can be obtained through participation in a standard global health academic program and cancer-specific competencies that can be obtained through an oncology training program. These competencies will be outlined and reviewed in detail in a subsequent publication.

### Review and Dissemination of the NCI/ASCO Survey on Global Oncology Research and Training Programs in NDCCs

Sixty-seven of 70 NDCCs (96%) responded to the survey. Thirty-three of the NDCCs reported having a global oncology program, and 61 reported a total of 613 non–NIH-funded global oncology projects.^18^ The survey identified diverse funding sources for global oncology activities, including external grants, institutional funding, and funding from other sectors. Of the 33 NDCCs with global oncology programs, half reported that trainees completed rotations outside the United States, and half enrolled trainees from LMICs. For NDCCs with a global oncology program, the majority reported having ≥ 11 faculty engaged in the program. The survey showed that the NDCCs are engaging in substantial and growing numbers of activities in global oncology.

## RECOMMENDATIONS

The AGOTF identified several action items to further develop global oncology as an academic discipline focused on improving cancer care and reducing cancer health disparities in resource-limited populations and settings, wherever they may occur. The recommendations were grouped into four categories: global oncology training, global oncology research and practice, global oncology career paths and professional development, and overall global oncology (Table 1). The final recommendations were presented to the ASCO Board of Directors in December 2018 and approved in February 2019.

**TABLE 1:**
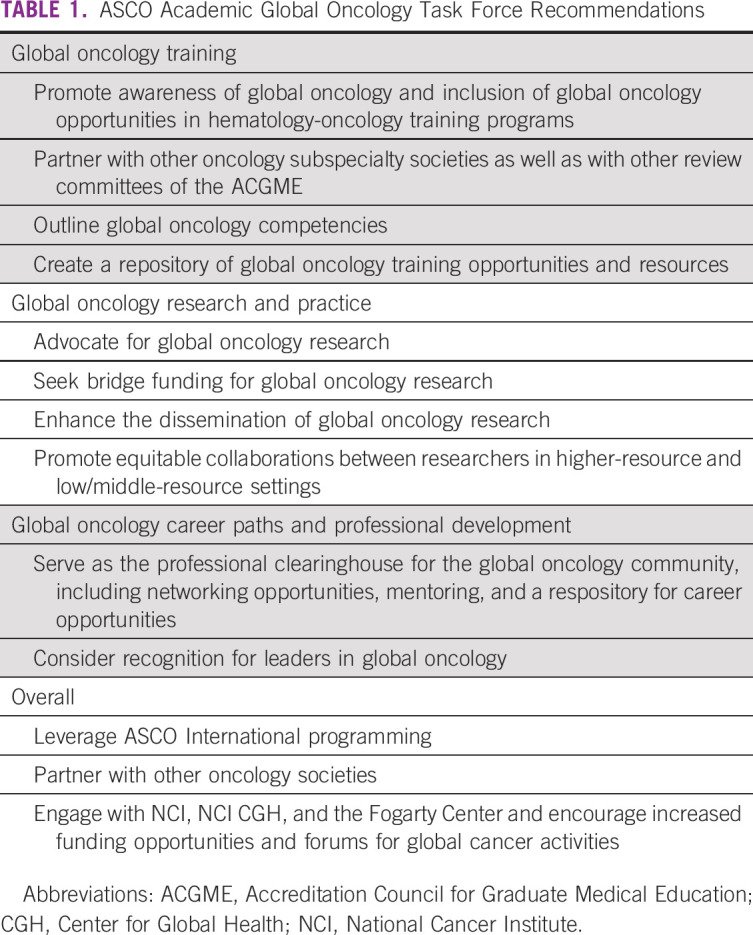
ASCO Academic Global Oncology Task Force Recommendations

### Global Oncology Training

#### Promote awareness of global oncology concepts and inclusion of global oncology opportunities in hematology-oncology training programs.

The AGOTF identified opportunities for innovation with regard to global oncology training within ACGME fellowship requirements. These opportunities for curricular flexibility are not currently well understood among training program directors. ASCO can provide valuable insights and guidance to training program directors to clarify the parameters of ACGME, facilitating trainee-friendly approaches to accommodating interest in international training and electives. For example, during the 2019 Annual Program Directors Retreat, this information was presented to training program directors who were in attendance, which represented one third of all domestic hematology-oncology fellowship training programs (57 of 167 ACGME-approved fellowship programs). In addition, this topic will be the focus of a 2020 Quarterly Connection call, in which domestic training program directors are invited to participate. This information will be shared on the training programs myConnection online forum so that all program directors can easily access it.

#### Partner with other oncology subspecialty societies as well as other review committees of the ACGME to determine whether the various subspecialty training programs have flexibility to offer global oncology opportunities to their trainees.

ASCO looks forward to sharing what it has learned with other specialties, such as the American College of Surgeons, Society of Surgical Oncology, American Society of Clinical Pathology, American Society for Radiation Oncology, American Society of Hematology, American College of Radiologists, Oncology Nursing Society, and other professional societies that represent key constituencies in the oncology workforce.

#### Outline competencies for those seeking to specialize in the field of global oncology.

The AGOTF’s proposed list of competencies consists of cancer-specific skills and knowledge over and above a standard oncology specialty training as well as global health skills and knowledge. These proposed global oncology competencies are distinct from, and build upon, a standard oncology training curriculum. It is envisioned that global health competencies would be provided by a standard global health training program, and the cancer-specific global oncology skills and knowledge would be provided by a select number of training programs that would specialize in this field. An advanced fellowship, like that offered for physicians training in blood and marrow transplantation, may offer the most practical and tailor-made way to provide in-depth global oncology training.

#### Serve as a repository for information on global oncology training opportunities and resources.

ASCO will work with the NCI CGH and other collaborators to create a repository of fellowship training programs that provide global oncology opportunities, facilitating the networking among these programs and the sharing of resources and opportunities. Such a repository could include resources for domestic and international training programs. ASCO could also promote interactions among trainees with an interest in global oncology through opportunities at the trainee lounge during the ASCO annual meeting.

### Global Oncology Research and Practice

#### Advocate for the relevance of global oncology research to understanding risk factors and cancer biology around the world and disparities and resource constraints in the United States as well as internationally.

Through publications and member communications, ASCO can emphasize that cancer research needs to improve outcomes for all of us, and global oncology research is a critical opportunity for bidirectional learning and discovery of solutions from all corners of the world. These communications must also emphasize that epidemiological and health services research, as well as implementation science, are as important as basic science and clinical research. ASCO should encourage the funding of organizations to support these efforts.

#### Lead by example by awarding bridge funding opportunities for global oncology research.

The development of a research career in global oncology requires years of sustained research activity and research support. ASCO and CCF have shown leadership in support of young global oncology investigators by funding the Global Oncology YIA. CCF is seeking continued funding for YIAs in global oncology and opportunities for a mechanism similar to the Career Development Award for awarding bridge funding to sustain these researchers and to keep people on the path.

#### Enhance the dissemination of global oncology research.

ASCO has shown leadership in the dissemination of global oncology research through the addition of a global health track in the education program of the annual meeting and the launch of JCO GO, both of which have substantially advanced the field. ASCO could build on this progress by asking the Scientific Program Committee to consider a global oncology track in the scientific program of the annual meeting and to be mindful of opportunities to reflect global oncology priorities in general plenary sessions.

#### Promote equitable and sustainable collaborations between researchers in higher-resource and low/middle-resource settings.

ASCO can serve a leading role in raising awareness of the importance of sustainable, win-win partnerships between higher-resource and lower-resource collaborators. Global oncology research funders and investigators in high-resource settings must be mindful of local partner research constraints, perspectives, and requirements. For example, protected time and salary support are critical issues for researchers and research centers in resource-limited settings. ASCO should encourage global oncology investigators and institutions in higher-resource settings to develop equitable relationships with counterparts in LMICs, including recognition of contributions. ASCO can help to develop research leaders in resource-limited settings who can identify key research topics related to cancer in their country/region. The International Development and Education Award (IDEA) and the International Innovation Grant are examples of ASCO’s commitment to supporting leadership and research in LMICs. These programs could further support academic global oncology by, for example, creating an annual meeting session on research collaborations between IDEA recipients and global oncology trainees from high-resource settings.

### Global Oncology Career Paths and Professional Development

#### Serve as the professional home for the global oncology community.

In line with ASCO’s other professional development programs and services, ASCO can serve as a forum for the global oncology community. ASCO will continue to host online fora, such as its Global Oncology Interest Group launched in 2018 through the ASCO myConnection web portal,^19^ and live fora, such as networking opportunities at the annual meeting. At the 2019 Annual Meeting, JCO GO provided mentoring opportunities to attendees interested in global oncology and continues to expand initiatives that match global oncology mentors with recent trainees interested in the field.

#### Consider recognition for leaders in global oncology.

ASCO can help to move the global oncology community forward by highlighting the exemplar leaders who are effectively supporting global oncology training in their fellowship programs, performing cutting-edge global oncology research, and improving the lives of patients in resource-limited settings. ASCO can consider opportunities to recognize the work of these trailblazing leaders in an emerging field.

### Overall

#### Leverage ASCO International programming to advance academic global oncology.

ASCO International programs can serve as a mechanism for disseminating global oncology research. Global oncology experts and junior faculty, including recipients of Global Oncology YIAs, should and will be engaged as ASCO International volunteers, including participation in ASCO International meetings, such as international palliative care courses, international clinical trials workshops, multidisciplinary cancer management courses, and cancer control in primary care courses. ASCO International programs can advance opportunities for academic global oncology when selecting sites and developing curricula and through encouragement of collaborations and ancillary on-the-ground activities. As ASCO develops new tools and programs, opportunities for global participation and support for global oncology should be considered.

#### Partner with other oncology societies to build sustained support for global oncology.

Numerous oncology-related professional societies are experiencing the same rise in interest in global oncology among their membership. ASCO will work with other oncology societies to coordinate activities in response to these AGOTF recommendations. Support for the continued development of global oncology as a viable academic discipline with a unique scope of leadership development will require a sustained and coordinated effort across the community.

#### Engage with NCI, NCI CGH, and the Fogarty Center to further the field of global oncology.

ASCO can make the case to the NCI that it should consider an increase in funding streams to support global cancer medicine research in the areas of implementation science, basic and translational science, and clinical science. NCI should consider a funding mechanism for oncology fellowship trainees through training grant mechanisms to support the development of future faculty in this area. The NCI should also consider further incorporating global oncology into the Cancer Center Support Grant (CCSG) guidelines for cancer centers, encouraging global efforts beyond the typical catchment area in the disparities/health equity section, to reinforce these efforts as an integral part of their applications.

## DISCUSSION

Global oncology is a necessary academic field to train experts who can think critically about innovative solutions to global cancer care and control and to provide them with the tools and support to conduct critical research and implement change. By publishing the recommendations of the AGOTF, we hope to generate action in addressing the challenges in formalizing global oncology as an academic discipline, building on ASCO’s expertise in supporting the professional development of its domestic and international members.

Success will require collaboration across all sectors of our global health and oncology communities and strong alliances with key stakeholders. Critical components for sustainable success in building the global oncology field will need to include the following:

- Demonstration of benefit to oncology training programs and academic cancer centers for supporting training in global oncology and specialization in academic global oncology as a career pathway
- Validation to funders of the necessity and value of supporting well-structured, evidence-focused research that will lead to improvements in cancer outcomes globally
- Establishment of strong partnerships with international health care providers and governments, patient advocates, governmental agencies, private philanthropic foundations, other professional organizations, and the business and corporate sector
- Promotion of responsibility in projects and partnerships, leveraging existing infrastructure and relationships, collaborating when reasonable and possible, and respecting international partners in terms of shared authorship, funding, and opportunities for their own career advancement^20,21^

With growing interest in global health among medical trainees, oncology training programs could benefit from supporting enhanced training in global oncology. Opportunities for international electives could make a program more attractive to prospective candidates and enrich the experience of trainees within the program. Oncology training programs would benefit by partnering with one another to increase trainee access to a variety of high-quality, off-site global oncology training experiences. The ACGME has restructured compliance requirements previously limiting global oncology opportunities by allowing flexibility with structure and oversight of continuity clinics for training programs in good standing, allowing an opportunity for increased international experiences during training.

Academic cancer centers will need to be convinced of their institution’s return on investment from faculty involvement in global oncology activities for the field to become a respected discipline. The NCI/ASCO survey shows that NDCCs are already engaged in substantial global oncology activity and that global oncology is indeed a desired career path for fellows and junior faculty.^18^ For academic cancer centers to see value in supporting careers and formal programs in global oncology, NIH funding could be a major driver. The current funding announcement for P30 CCSGs for NDCCs mentions the word global in only two sections: Cancer Research Training and Education and Community Outreach and Engagement.^22^ The NCI could increase cancer center director interest in supporting global oncology training and research through a stronger emphasis in the CCSG evaluation process. In addition to NIH funding opportunities, the NCI/ASCO survey of NDCCs revealed many global oncology projects supported by diverse, non-NIH funding sources, identifying additional funders of global cancer research for potential further engagement.^19^

Academic careers in global health have unique challenges, including issues in meeting requirements for clinical service requiring onsite continuity care, limited opportunities for conventional research funding, and difficulty in meeting traditional metrics for promotion and academic advancement.^8^ For faculty to be successful in pursuing an academic career in global oncology, institutions will need to be flexible and innovative in structuring clinical care requirements and develop metrics that reflect valuable academic contributions, including models for mentorship, outside the conventional promotion pathway.^23,24^

ASCO strives to better understand how to enhance its engagement at a global level. ASCO has committed to providing a forum for global oncology research through JCO GO and by including a formal global health track at the ASCO annual meeting. ASCO’s CCF supports a multitude of opportunities for professional development and research in global oncology, including IDEAs, international innovation grants, and the Global Oncology YIA. In response to the recommendations from the AGOTF, ASCO will be developing and implementing additional opportunities to further support formal and robust global oncology training, career development, and research.

There is much work to be done to establish global oncology as a structured, recognized, and respected academic discipline. The leadership, volunteers, and membership of ASCO look forward to collaborating with stakeholders across all sectors of the global health and oncology communities in realizing the vision of equity and improved cancer outcomes worldwide.
